# A Microfluidic Chip for Single-Cell Capture Based on Stagnation Point Flow and Boundary Effects

**DOI:** 10.3390/mi15040456

**Published:** 2024-03-28

**Authors:** Long Cheng, Xiao Lv, Wenchao Zhou, Huan Li, Qiushuang Yang, Xing Chen, Yihui Wu

**Affiliations:** 1 Changchun Institute of Optics, Fine Mechanics and Physics, Chinese Academy of Sciences, Changchun 130033, China; chenglong211@mails.ucas.ac.cn (L.C.); lvxiao@ciomp.ac.cn (X.L.); yangqiushuang666@163.com (Q.Y.); chenxing222@mails.ucas.ac.cn (X.C.); yihuiwu@ciomp.ac.cn (Y.W.); 2 University of Chinese Academy of Sciences, Beijing 101408, China; 3 State Key Laboratory of Applied Optics, Changchun 130033, China; 4 Key Laboratory of Optical System Advanced Manufacturing Technology, Chinese Academy of Sciences, Changchun 130033, China

**Keywords:** microfluidic chip, single-cell capture, stagnation point flow, boundary effects

## Abstract

The capture of individual cells using microfluidic chips represents a widely adopted and efficient approach for investigating the biochemical microenvironment of singular cells. While conventional methods reliant on boundary effects pose challenges in precisely manipulating individual cells, single-cell capture grounded in the principle of stagnation point flow offers a solution to this limitation. Nevertheless, such capture mechanisms encounter inconsistency due to the instability of the flow field and stagnation point. In this study, a microfluidic device for the stable capture of single cells was designed, integrating the principle of fluid mechanics by amalgamating stagnation point flow and boundary effects. This innovative microfluidic chip transcended the limitations associated with single methodologies, leveraging the strengths of both stagnation point flow and boundary effects to achieve reliable single-cell capture. Notably, the incorporation of capture ports at the stagnation point not only harnessed boundary effects but also enhanced capture efficiency significantly, elevating it from 31.9% to 83.3%, thereby augmenting capture stability. Furthermore, computational simulations demonstrated the efficacy of the capture ports in entrapping particles of varying diameters, including 9 μm, 14 μm, and 18 μm. Experiment validation underscored the capability of this microfluidic system to capture single cells within the chip, maintaining stability even under flow rate perturbations spanning from 60 μL/min to 120 μL/min. Consequently, cells with dimensions between 8 μm and 12 μm can be reliably captured. The designed microfluidic system not only furnishes a straightforward and efficient experimental platform but also holds promise for facilitating deeper investigations into the intricate interplay between individual cells and their surrounding microenvironment.

## 1. Introduction

Currently, the study of single-cell behavior and analysis, particularly the intricate interplay between individual cells and the microenvironment, has emerged as a prominent research focus within the field of biology [1,2,3]. This area of inquiry assumes pivotal significance in various applications, including single-molecule detection [4,5], cell sorting [6,7], single-cell sequencing [8], and investigations into physiological and pathological cellular processes [9]. Wu et al. discussed electric field, mechanical, and fluorescence-based single-cell analysis and characterization methods [10]. Kulkarni et al. discussed the study of miniaturized biosensors for the detection of nucleic acid biomarkers [11].

Single-cell capture constitutes the pivotal initial phase in conducting single-cell analysis. To date, microfluidic technology, distinguished by its unique advantages over traditional analytical approaches in cell analysis, has emerged as a prominent and efficient platform for conducting single-cell capture experiments [12]. Microfluid systems offer tailored solutions to the requirements of single-cell analysis, boasting advantages such as precise control over reagent volumes, streamlined cell processing capabilities, and seamless integration with automation systems. It is of great significance in the fields of medical diagnosis, health detection, and food safety. Various fabrication methods have been developed for microfluidic chips. Kulkarni et al. used a microfluidic device to perform the on-site detection of real-time DNA amplification and developed an ocular drug delivery system using a microfluidic device with 3D printing technology [13,14]. Yu et al. used a microfluidic chip bonded with PDMS and glass to perform biochemical stimulation on single cells [15]. In previous investigations, diverse methodologies have been proposed for capturing single cells within microfluidic chips, including electrodynamic trapping [16], magnetic trapping [17], optical trapping [18], and hydrodynamic capture [19]. These methodologies have been extensively employed to explore an array of both physical and biological phenomena.

In contemporary microfluidics, two primary hydrodynamic principles are utilized for single-cell capture: contact and non-contact. Contact microfluidics employ boundary barriers within the channels to ensnare particles or cells traversing the microfluidic system. While these contact-based approaches facilitate the rapid sequential capture of a substantial number of single cells, often arranged in series or with arrays, they commonly encounter challenges associated with achieving stable single-cell capture [20]. An additional method entails non-contact principles. Non-contact capture is represented by the principle of stagnation point flow. Non-contact flow channels exhibit the capability for the delicate manipulation of single cells and facilitate liquid-to-liquid exchange. However, they typically fall short in achieving the stable capture of large numbers of single cells arranged in arrays [21]. The incorporation of external sensors can enhance operability, yet it necessitates the utilization of intricate and costly acoustic or optical sensors to ensure the consistency of stagnation spot flow or micro-vortex capture [22]. Dockx et al. investigated the effect of geometric parameters on the 3D flow field through numerical simulation and found that the resulting stagnation point flow and micro-vortex have the ability to capture single cells [23], but it was limited to the simulation analysis of the fluid as well as fluid flow experiments and was not verified by actual capture experiments on microparticles. Dang et al. proposed a dual hysteresis microfluidic model that can capture and control two microparticles at the same time. They optimized the generation and stagnation points of the vortex region and characterized the velocity and streamlines [24]. However, stable capture remains challenging due to the inherent instability of the flow field and the stationary point.

To attain stable single-cell capture, we developed a microfluidic chip leveraging the principles of stagnation point flow and boundary effects. Compared to alternative techniques, the technique described herein offers distinct advantages. The advantage is that the cell is first decelerated to the vicinity of the capture port using the principle of stagnation point flow, and then the cell is stably captured at the capture port due to the boundary effect of the capture port. After capture, the resistance channel closes, and a stagnation point of zero flow velocity is formed at the capture port. The combination of the two enables the stable capture of single cells, and the design of the capture port enables the capture of cells of different sizes. It facilitates the isolation of single cells of specific sizes and ensures their stable positioning at the capture point. This meticulously designed microfluidic chip interfaces with a programmable syringe pump, a high-powered microscope, and an imaging computer, thereby constituting a comprehensive microfluidic system. The efficacy of the chip will be validated through integrated system experiments aimed at achieving consistent single-cell capture.

## 2. Materials and Methods

### 2.1. Microfluidic Channel Theory with Stagnation Point

The microfluidic channel within the designed microfluidic chip exhibits a high aspect ratio, with the transverse width (W) significantly smaller than the length (L). Flow within the channel predominantly adopts a laminar state. Consequently, a method akin to that employed in dealing with plane potential flow is applicable, wherein complex potentials are introduced in the plane, and the average flow velocity is obtained according to the potential function and flow function.

This entails introducing the complex potential $W\left(Z\right)=\varphi \left(r,\theta \right)+j\psi \left(r,\theta \right)=AZ^{n}$, where $Z=re^{j\theta }$, A is a real number, n is a positive number greater than 1, and $j=\sqrt{\left(-1\right)}$. Through this approach, the average flow velocity $\overset{-}{V}\left(r,\theta \right)$ can be expressed as a function of potential $\varphi \left(r,\theta \right)$ and the flow function $\psi \left(r,\theta \right)$ [25,26]:

(1) $$\overset{-}{V}\left(r,\theta \right)=\frac{\partial \varphi }{\partial r}e_{r}+\frac{1}{r}\frac{\partial \varphi }{\partial \theta }e_{\theta }=\frac{1}{r}\frac{\partial \psi }{\partial \theta }e_{r}-\frac{\partial \psi }{\partial r}e_{\theta }={\mathrm{Anr}}^{n-1}[\cos\left(n\theta \right)e_{r}-\sin\left(n\theta \right)e_{\theta }]$$

where $e_{r}$ and $e_{\theta }$ denote the basis vectors in polar coordinates.

Assuming constancy in the potential function and flow function, contours and flow lines can be delineated when n = 2.5. At the origin of coordinates, the average flow velocity $\overset{-}{V}\left(r,\theta \right)$ is zero, indicating the fluid stagnation point.

Subsequently, a microchamber capture section featuring a fluid stagnation point is constructed, with its symmetry axis aligned along the *X*-axis. The upper and lower boundary curves coincide with two symmetrical flow lines, satisfying the following boundary equations [25].

(2) $$r^{n}\sin\left(n\theta \right)=r_{0}^{n}\sin\left(n\theta_{0}\right)\;\;(\theta \in [\theta_{0},\pi -\theta_{1}])$$

and

(3) $$r^{n}\sin\left(n\theta \right)=-r_{0}^{n}\sin\left(n\theta_{0}\right)\;\;\left(\theta \in \left[\theta_{1}-\pi ,-\theta_{0}\right]\right)$$

where $\theta_{1}$ represents the complementary angle of the polar angle associated with the upper curve boundary. Consequently, when n = 2.5 [27], $\theta_{1}=3/4\pi$. Additionally, $\theta_{0}$ and $r_{0}$ denote the angle and pole diameter, respectively, of the starting point of the curve boundary on the flow chamber within the (x,y) plane, as represented by

(4) $$\theta_{0}\approx \tan^{-1}\frac{W}{2L}$$

and

(5) $$r_{0}=\sqrt{L^{2}+\frac{W^{2}}{4}}$$

**Figure 1:**
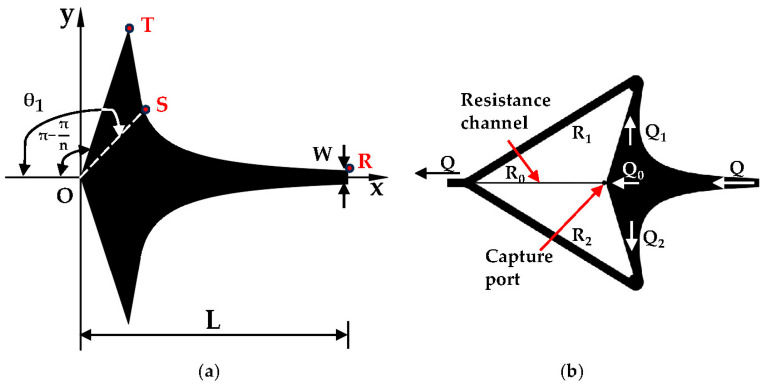
(**a**) RS represents the curve boundary, while ST and TO denote the straight-line boundaries. (**b**) The capture chamber system mechanism.

In the equation, depicted in Figure 1a, L denotes the total length of the flow chamber channel, while W represents the inlet width of the flow chamber. Subsequently, two additional flow lines passing through the coordinate origin are chosen to constitute part of the boundary of the flow chamber. The linear equation governing the upper and lower boundaries is determined as follows:

(6) $$\theta =\pm \frac{\pi }{n}(r\in \left[0,\frac{L}{2}\right])$$

For this flow chamber, the equation for its depth-averaged flow velocity along the central axis X is determined as follows [25]:

(7) $$\left|\overset{-}{V}\left(r,0\right)\right|=\frac{{\mathrm{Qnr}}^{n-1}}{2Hr_{0}^{n}\sin\left(n\theta_{0}\right)}$$

where Q denotes the total flow rate entering the flow chamber from the inlet.

### 2.2. The Combination of the Boundary Effect, Stagnation Point Flow, and Resistance Channel

According to the flow velocity distribution within the flow chamber and the plane potential flow theory outlined in Section 2.1, the following observations can be derived: along the central axis of the flow chamber, the polar diameter r gradually diminishes, accompanied by a progressive reduction in the average flow velocity from its maximum value at the inlet, ultimately reaching zero as per Equation (7). Theoretically, if a cell enters the flow chamber through the inlet and travels along the axis towards the fluid stagnation point (i.e., the cell capture point located at the coordinate origin), it encounters a zero flow rate, facilitating stable entrapment. However, due to the influence of side boundaries and fluid viscosity, achieving easy and stable cell entrapment at this juncture proves challenging despite the absence of flow. Consequently, the capture chamber is devised with consideration for cell size at the capture port. This entails the design of a resistance channel at the rear, as depicted in Figure 1b, supplemented by two output channels, upper and lower, facilitating cell traversal towards the capture port while mitigating the risk of escape.

The principle of minimum flow resistance for single-cell capture necessitates that the combined flow resistance of the main channel, denoted as $R_{1}$+$R_{2}$, exceeds the flow resistance of the capture channel, denoted as $R_{0}$. As per the Darcy–Weisbach law applied to a rectangular channel [28], the following expression is obtained:

(8) $$∆p=\frac{C(\alpha )}{32}\frac{{\mu \mathrm{KQP}}^{2}}{A^{3}}$$

(9) Cα=f×Re=96×(1−1.3553×α+1.9467 × α2−1.7012 × α3+0.9564 × α4−0.2537 × α5)

(10) $$\frac{Q_{0}}{Q_{1}+Q_{2}}=\frac{2C(\alpha_{1})}{C(\alpha_{0})}\times {\left(\frac{A_{0}}{2A_{1}}\right)}^{3}\times {\left(\frac{2P_{1}}{P_{0}}\right)}^{2}\times \left(\frac{{2K}_{1}}{K_{0}}\right)>1$$

where ∆p represents the pressure difference between the inlet and outlet, while Q signifies the total volume flow; A and P denote the cross-sectional area and perimeter of the channel, respectively. K and μ represent the path length and fluid viscosity, respectively. Furthermore, C(α) denotes the product of the Darcy friction factor f and the Reynolds number Re.

### 2.3. Flow Chamber Simulation

The flow velocity characteristics of the designed flow chamber were simulated using COMSOL Multiphysics 5.6. The 3D model, imported from AutoCAD, was extended to a depth of 60 μm, with liquid water specified as the material. Fluid properties were derived based on laminar flow characteristics, with appropriate physics incorporated. Boundary conditions were established to ensure no slippage at the sidewalls, while maintaining a normal inflow velocity ranging from 60 μL/min to 120 μL/min at the inlet cross-section and implementing outlet conditions to prevent reflux. Following mesh refinement, convergence criteria were applied, and the simulation was conducted under steady-state conditions, ultimately resolving the laminar flow physics.

**Figure 2:**
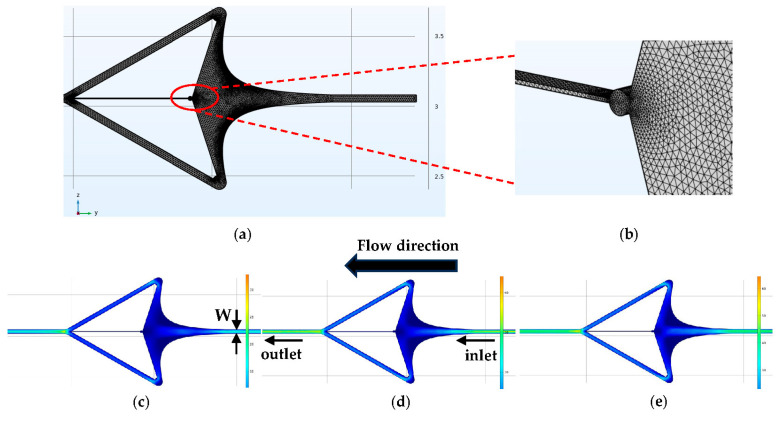
Flow velocity profile. (**a**) Meshing diagram. (**b**) View of capture port. (**c**) Inlet flow rate of 60 μL/min. (**d**) Inlet flow rate of 80 μL/min. (**e**) Inlet flow rate of 120 μL/min.

In Figure 2, the flow velocity distribution within the flow chamber exhibits notable symmetry, with a gradual decrease observed along the flow direction. Importantly, the flow velocity at the cell capture point approaches zero, affirming the presence of a stagnation point. Furthermore, varying the flow rate maintains this velocity pattern. Notably, in terms of flow behavior, velocity is deflected near the stagnation point, resulting in flow diversion towards the upward and downward exit directions. Consequently, when a single cell is captured and the resistance channel is obstructed, other cells are directed out of the output channel along the trajectory of flow lines, thereby facilitating single-cell capture.

The design of a capture port at the stagnation point is motivated by two primary objectives. Firstly, it enables the amalgamation of stagnation point flow and boundary effects, thereby enhancing the stability of single-cell capture. Secondly, the diameter of the capture port can be tailored according to the size of the targeted cell. This customization serves to prevent interference from additional cells, ensuring that only one cell enters the capture point at a time and thereby maintaining capture stability. Moreover, this approach facilitates investigations into how cell size influences capture efficiency. Such analyses contribute to a deeper understanding of how specific cells can be targeted, manipulated, and analyzed using these devices.

**Figure 3:**
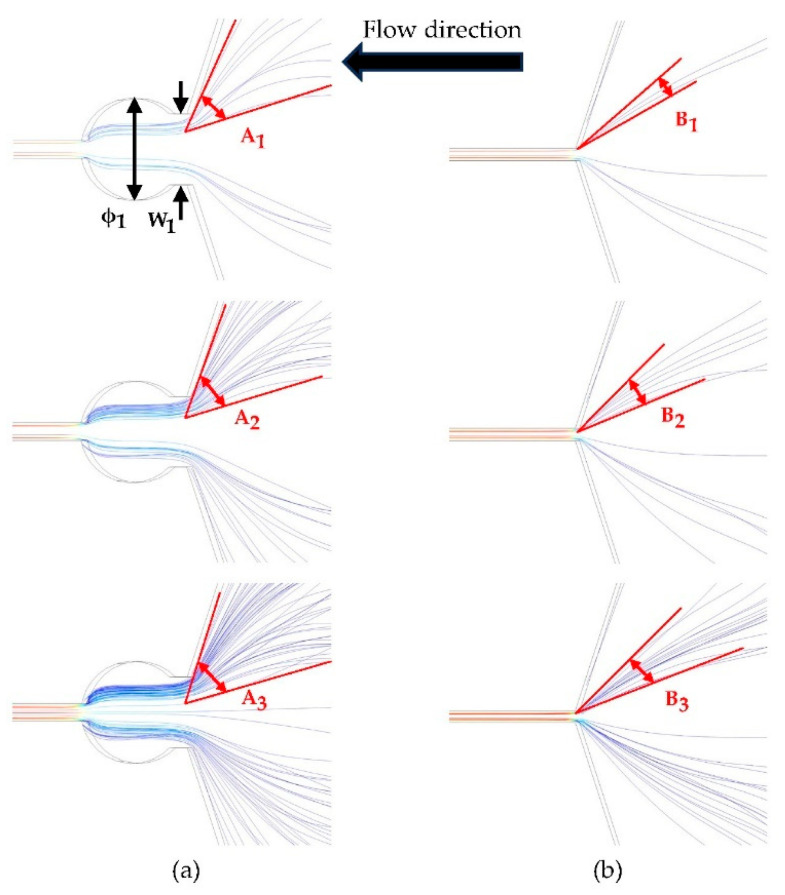
Streamline comparison diagram. (**a**) Three streamlines with circular capture ports. (**b**) Three streamlines without circular capture ports.

In Figure 3, the streamlines of the two capture ports were simulated under inlet flow rates of 60 μL/min, 80 μL/min, and 120 μL/min, respectively. As the flow velocity increases, the number of streamlines passing through the capture port also increases. However, the increment of streamlines in the central axis region is not pronounced, indicating the presence of a stagnation point at the capture point, where the flow velocity approaches zero. Notably, the streamlines reveal that cells tend to be trapped in proximity to the stagnation point, a phenomenon which warrants validation through subsequent experiments. A comparison of the streamline diagrams with and without the circular capture port reveals distinct differences. Specifically, the streamline pattern with the circular capture port appears more scattered, indicative of a larger capture area. Introducing a circular capture port results in smoother streamlines, facilitating ease of cell entrapment. Conversely, the streamline gradient acceleration without a circular capture port rises too abruptly at the stagnation point, posing the risk of cell rupture and impeding stable capture.

In Figure 3a, the parameters for the capture port are as follows:$\;\phi_{1}$ is 15 μm. $W_{1}$ is 12 μm. $A_{1}$, $A_{2},$ and $A_{3}$ are 50°, 55°, and 60°, respectively, resulting in a total capture port angle of 144°. By dividing the angle of 60° by the total capture port angle of 144°, the calculated capture efficiency is 83.3%. Conversely, the parameters for the structure without a capture port in Figure 3b are 11°, 20°, and 23° for $B_{1}$, $B_{2}$, and $B_{3}$, respectively, and the resulting capture efficiency is only 31.9%. From the above data, it can be inferred that the flow chamber equipped with a capture port exhibits a larger capture area, thereby facilitating a more straightforward achievement of stable cell capture.

**Figure 4:**
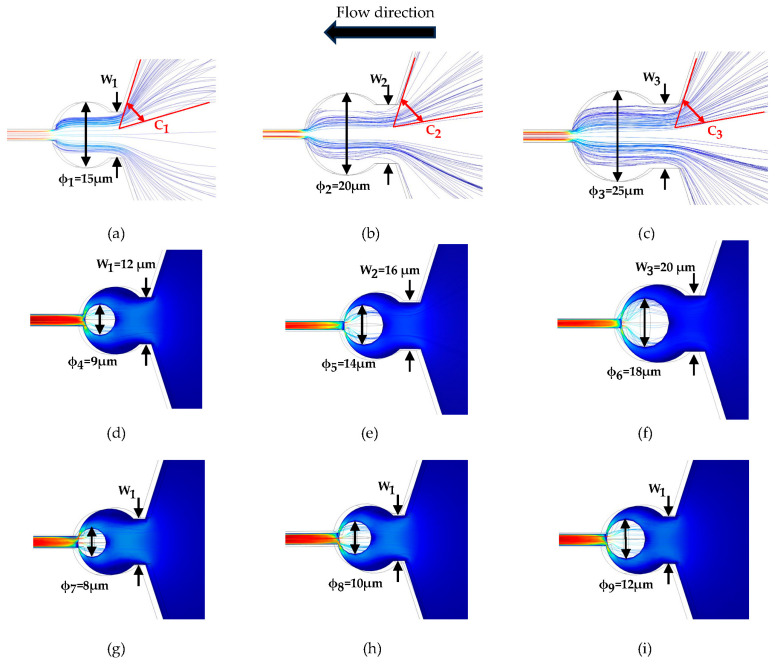
(**a**–**c**) Simulation of streamlines with different diameters ($\phi_{1}$ = 15 μm, $\phi_{2}$ = 20 μm, and $\phi_{3}$ = 25 μm). (**d**–**f**) Simulation of different inlet widths ($W_{1}$ = 12 μm, $W_{2}$ = 16 μm, and $W_{3}$ = 20 μm) for capturing microparticles of different diameters ($\phi_{4}$ = 9 μm, $\phi_{5}$ = 14 μm, and $\phi_{6}$ = 18 μm). (**g**–**i**) Simulation of capture of microparticles of different diameters ($\phi_{7}$ = 8 μm, $\phi_{8}$ = 10 μm, and $\phi_{9}$ = 12 μm) with same inlet width ($W_{1}$ = 12 μm).

In Figure 4, $W_{1}$, $W_{2}$, and $W_{3}$ are 12 μm, 16 μm, and 20 μm, respectively, and the streamline simulation is performed at the same flow rate of 120 μL/min. $C_{1}$, $\;C_{2}$, and $C_{3}$ are 60°, 61°, and 62°, respectively. Capturing particles or cells of varying sizes plays a pivotal role in effectively separating, analyzing, or processing entities within a specific size range. Employing capture devices featuring capture ports of different diameters enables distinct capture performances tailored to particles or cells of varying diameters. This approach facilitates the effective capture of particles or cells across a spectrum of sizes. As evidenced by Figure 4a–c, the streamline patterns persist even after increasing the diameter of the capture port. Subsequently, Figure 4d–f indicate that capture ports with different apertures are capable of capturing cells or particles of different particle sizes, thus affirming their ability to capture single cells across a range of sizes. Finally, Figure 4g–i indicate that a capture port with an inlet width of 12 μm and a diameter of 15 μm is capable of capturing microparticles of 8–12 μm.

### 2.4. Design and Fabrication of Microfluidic Chip

**Figure 5:**
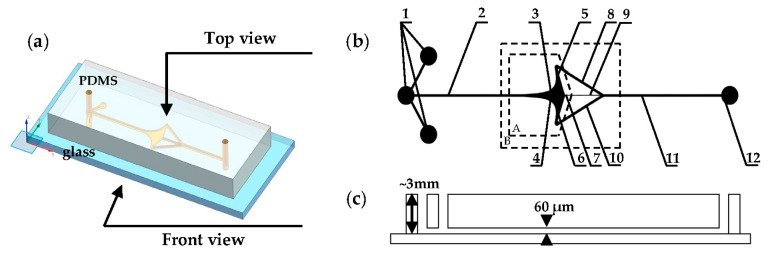
(**a**–**c**) The design of the PDMS–glass-bonded microfluidic chip. The chip design is delineated as follows: A represents the capture chamber, while B represents the capture microfluidic channel system. The components are labeled as follows: 1—fluid injection inlets, 2—cell suspension inlet channel, 3—upper boundary curve, 4—lower boundary curve, 5—upper straight-line boundary, 6—lower straight-line boundary, 7—stagnation point capture port, 8—upper outlet channel, 9—resistance channel, 10—lower outlet channel, 11—cell suspension outlet channel, and 12—fluid outlet.

In Figure 5, The microfluidic chip utilized in this study is a polydimethylsiloxane and glass (PDMS–glass)-bonded chip, with a consistent channel height of 60 μm across all channels and the capture chamber. Upon selecting n = 2.5, the angle between the upper- and lower-line boundaries is determined to be 144°, as detailed in Equation (6). Given that most cells range between 8 and 12 μm in diameter, the inlet width (W) of the single-cell capture chamber is set at 50 μm, with a length (L) of 1 mm. To ensure stable capture, a capture circle with a diameter of 15 μm is designed at the stagnation point. The width of the resistance channel $W_{r}$ is specified as 5 μm. Additionally, the widths of the remaining channels are consistent with the inlet widths, all measuring 50 μm. The comprehensive set of parameters defining the chip is provided in Table 1.

Once the overall design of the chip is finalized, it undergoes several processing steps including mask preparation, photolithography, etching, mold inversion, and chip bonding. Given that the minimum line width of the designed microfluidic channel is 5 μm, the pattern is transferred onto a chrome-plated mask. A layer of positive photoresist (AZ5214, China) is then coated onto a 3-inch double-polished silicon wafer. Subsequently, the silicon wafer is exposed using a lithography machine (Karl Suss MA6/BA6, Germany) with a chromium plate, followed by development. The silicon wafer is then subjected to inductively coupled plasma etching (Alcatel 601E ICP, France) to achieve an etching depth of 60 μm. The configured PDMS (Sylgard 184, China) and curing agent liquid are poured onto the silicon wafer, followed by curing at a temperature of 80 °C for 3 h. The PDMS mold is then demolded from the silicon wafer, and the molding process is repeated. Once a PDMS chip with fluid pathways is obtained, it undergoes punching for oxygen plasma bonding with cleaned glass.

### 2.5. Cell Suspension Preparation

The SIHA cell line was procured from Delta Biologics, Changchun, China. RPMI-1640 (Roswell Park Memorial Institute medium 1640), fetal bovine serum (FBS), phosphate-buffered saline (PBS), trypsin-EDTA, and penicillin/streptomycin were purchased from Baijin Biologics, Changchun, China.

The SIHA cells were cultured in RPMI-1640 medium supplemented with 10% FBS and 2% penicillin/streptomycin bispecific antibody. Following the fifth generation of cell culture in standard tissue culture medium, cells were harvested using trypsin, followed by centrifugation. Subsequently, a new cell culture medium was added to reconfigure into a PBS cell suspension, achieving a density of approximately 10^6^ cells/mL. It is noteworthy that the majority of cells exhibited diameters ranging from 8 to 12 μm.

### 2.6. Construction of Microfluidic Integrated System

**Figure 6:**
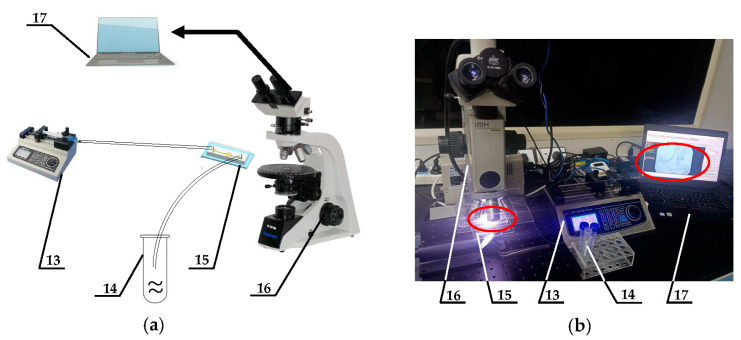
(**a**) The experimental schematic diagram of the microfluidic system. (**b**) The experimental platform construction diagram. The detailed description of the system is as follows: 13—fluid drive systems with cell suspension, 14—waste, 15—microfluidic chips, 16—imaging microscopes, 17—computer display systems.

In Figure 6, the microfluidic chip is integrated with a programmable syringe pump (LONGER, UK) and an imaging microscope equipped with a high-speed camera (FASTCAM UX100, Japan), constituting a comprehensive single-cell capture microfluidic system. The dosing syringe, propelled by the programmable syringe pump, regulates the flow and velocity of the fluid injected into the microfluidic inlet. Additionally, the computer is linked to a high-speed camera, facilitating the real-time imaging and display of captured images via computer software (Photron FASTCAM Viewer Ver.3680). First, an injection rate of 60 μL/min was employed to validate the stability of the structure for single-cell capture. Subsequently, a capture experiment involving flow field disturbance was conducted to confirm the system’s capture stability. This involved modifying the flow rate of the syringe pump to 60 μL/min and 120 μL/min, respectively.

## 3. Results

### 3.1. Single-Cell Capture Experiments

**Figure 7:**
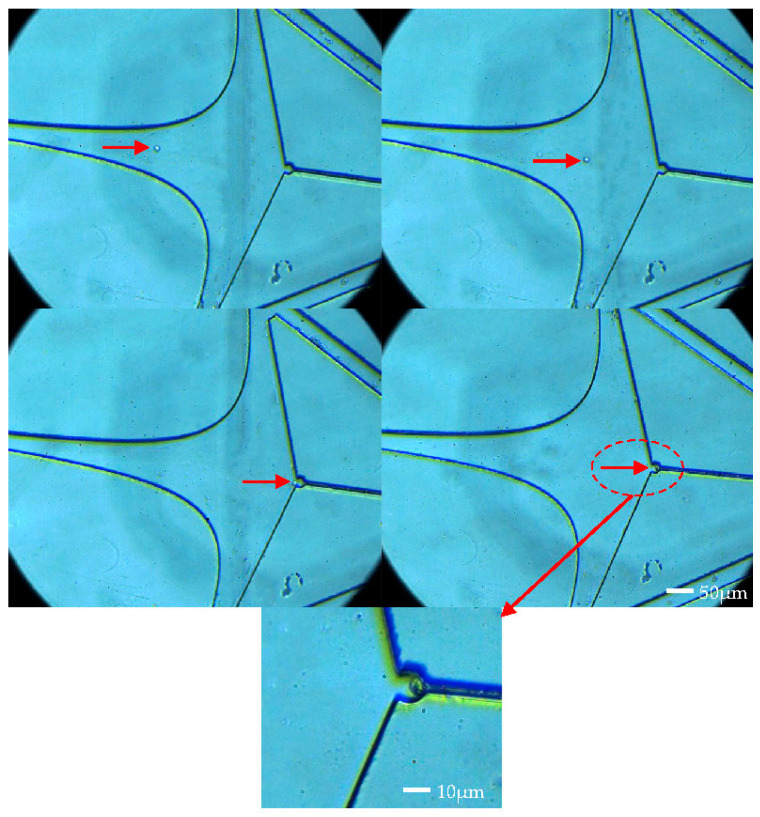
Capture trajectories of individual SIHA cell.

Experiments were initially conducted at an injection rate of 60 μL/min. In Figure 7, cells marked with red arrows were observed entering the capture chamber along the axis from its entrance. Notably, at the front and middle segments of the capture chamber, the influx rate was notably higher. However, towards the rear end of the capture chamber, a deceleration effect was observed, resulting in a gradual reduction in movement speed. As the cells approached the capture point, they did not decelerate directly into the capture port. Instead, they deviated at a certain angle before entering the capture port. This observation aligns with the streamlined results obtained from simulations. Cell trapping is not solely attributed to sidewall blockage, rather, it is a synergistic result of both zero flow rates and a physical barrier located at the boundary of the capture port.

**Figure 8:**
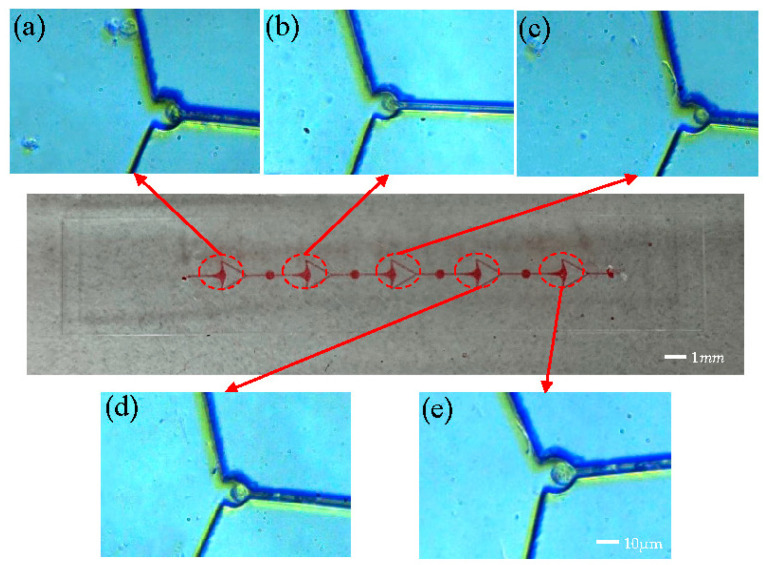
Array microfluidic chip capture experiment. (**a**–**e**) Capture of 5 capture ports in sequence.

Figure 8a–e depict the single-cell capture diagram of an array microfluidic chip comprising five units interconnected at the beginning and end. Experiments were conducted directly at an injection rate of 120 μL/min, resulting in cell capture at all five capture ports. Notably, the sizes of the captured cells varied across the ports, with the cell at the third capture port (Figure 8c) measuring approximately 8 μm, while the cell at the fifth capture port (Figure 8e) was approximately 12 μm. The observation of the captured cells reveals noteworthy distinctions. For instance, the first capture port of the array chip (Figure 8a) displays significant cell deformation attributable to the high flow velocity, with cells being nearly squeezed into the resistance channel. Conversely, the final capture port (Figure 8e) exhibits a lower flow rate, resulting in cells with normal morphology.

Based on the experimental observations, the following conclusions can be drawn. Conclusion 1: The array of flow chambers within the same unit demonstrates the capability to capture single cells. With the progression of liquid flow, the flow velocity gradually decreases within the subsequent capture chamber. Ultimately, even the final capture port retains the ability to capture single cells. Conclusion 2: The capture port exhibits the capacity to capture cells of varying diameters. This capability arises from the design specifications. Consequently, cells ranging from larger than 8 μm to less than 12 μm can be effectively captured.

### 3.2. Stable Capture under Flow Disturbances

**Figure 9:**
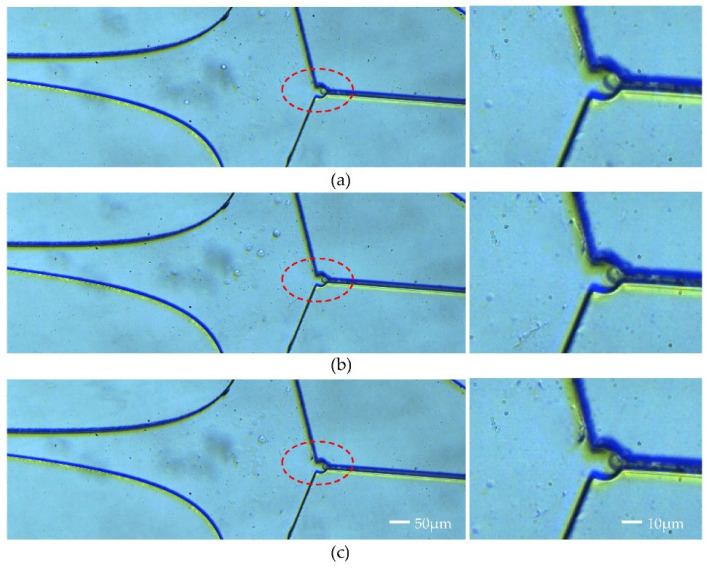
(**a**–**c**) Single-cell capture under flow perturbations.

After capturing a single SIHA cell, the inlet flow rate was incrementally raised from 60 μL/min to 120 μL/min. In Figure 9, the cell remained stable at the capture point, with the red-marked section being clearly discernible upon magnification. At an inlet flow rate of 60 μL/min, the captured cell exhibited normal morphology without fully contacting the capture wall (Figure 9a). Upon increasing the inlet flow rate to 80 μL/min, a slight deformation of the trapped cell was observed, with a small portion being pushed into the resistance channel (Figure 9b). Subsequently, at a flow rate of 120 μL/min, a significant deformation of the cells occurred due to elevated flow rate pressure; however, the cells remained intact (Figure 9c). By comparing the scenarios depicted in Figure 9, it was demonstrated that despite a substantial increase in flow rate, leading to a considerable perturbation of the flow field, the trapped cells remained stably positioned at the capture port.

## 4. Conclusions

To achieve stable single-cell capture, both the resistance channel and the capture port play pivotal roles. Consequently, cells ranging in size from larger than 8 μm to less than 12 μm can be effectively captured. It is worth noting that trapping is not solely attributed to sidewall blockage but rather by a combination of zero flow velocity and a physical barrier at the capture port boundary.

In summary, this study introduces a microfluidic chip that combines the principles of stagnation point flow and boundary effects to achieve single-cell capture. The incorporation of capture ports at the stagnation point not only merges boundary effects but also significantly enhances capture efficiency, increasing it from 31.9% to 83.3%, hereby enhancing capture stability. In addition, simulations demonstrated the capture port’s capability to capture particles with diameters of 9 μm, 14 μm, and 18 μm for different sizes. Experiments confirmed the microfluidic system’s ability to capture single cells within the chip at a flow rate of approximately 60 μL/min and stable capture at perturbations from 60 μL/min to 120 μL/min. The microfluidic device exhibits stable and efficient single-cell capture, even in the presence of significant flow disturbances. However, challenges such as high machining accuracy, multifunctional integration limitations, and cell clogging issues for deformable cells persist. Addressing these challenges will be a primary focus of our future research endeavors. Despite these challenges, the designed microfluidic system offers a straightforward and effective experimental platform for further exploring the intricate relationship between single cells and their dynamic biochemical microenvironment.

## Figures and Tables

**Table 1:** The main design parameters and fluid experimental parameters.

Parameters	Values
n	2.5
$\theta_{1}$	3/4π
Cell diameter	8~12 μm
$W_{1}$	12 μm
$\mathrm{The}\;\mathrm{diameter}\;\mathrm{of}\;\mathrm{the}\;\mathrm{capture}\;\mathrm{port}\;\phi_{1}$	15 μm
Length L (*x*-direction)	1 mm
The width of the inlet W (*y*-direction)	50 μm
Height H (*z*-direction)	60 μm
$\mathrm{The}\;\mathrm{width}\;\mathrm{of}\;\mathrm{the}\;\mathrm{resistance}\;\mathrm{channel}\;W_{r}$	5 μm
The rest of the channel widths	50 μm
Inlet flow rate	60 μL/min, 80 μL/min, and 120 μL/min

## Data Availability

The original contributions presented in the study are included in the article, further inquiries can be directed to the corresponding authors.
